# Primary sclerosing cholangitis and the path to translation

**DOI:** 10.1172/JCI174218

**Published:** 2023-09-01

**Authors:** Lóránd Váncza, Natalie J. Torok

**Affiliations:** 1 Gastroenterology and Hepatology, Stanford University, Stanford, California, USA.; 2 VA, Palo Alto, California, USA.

## Introduction

**Figure 1:**
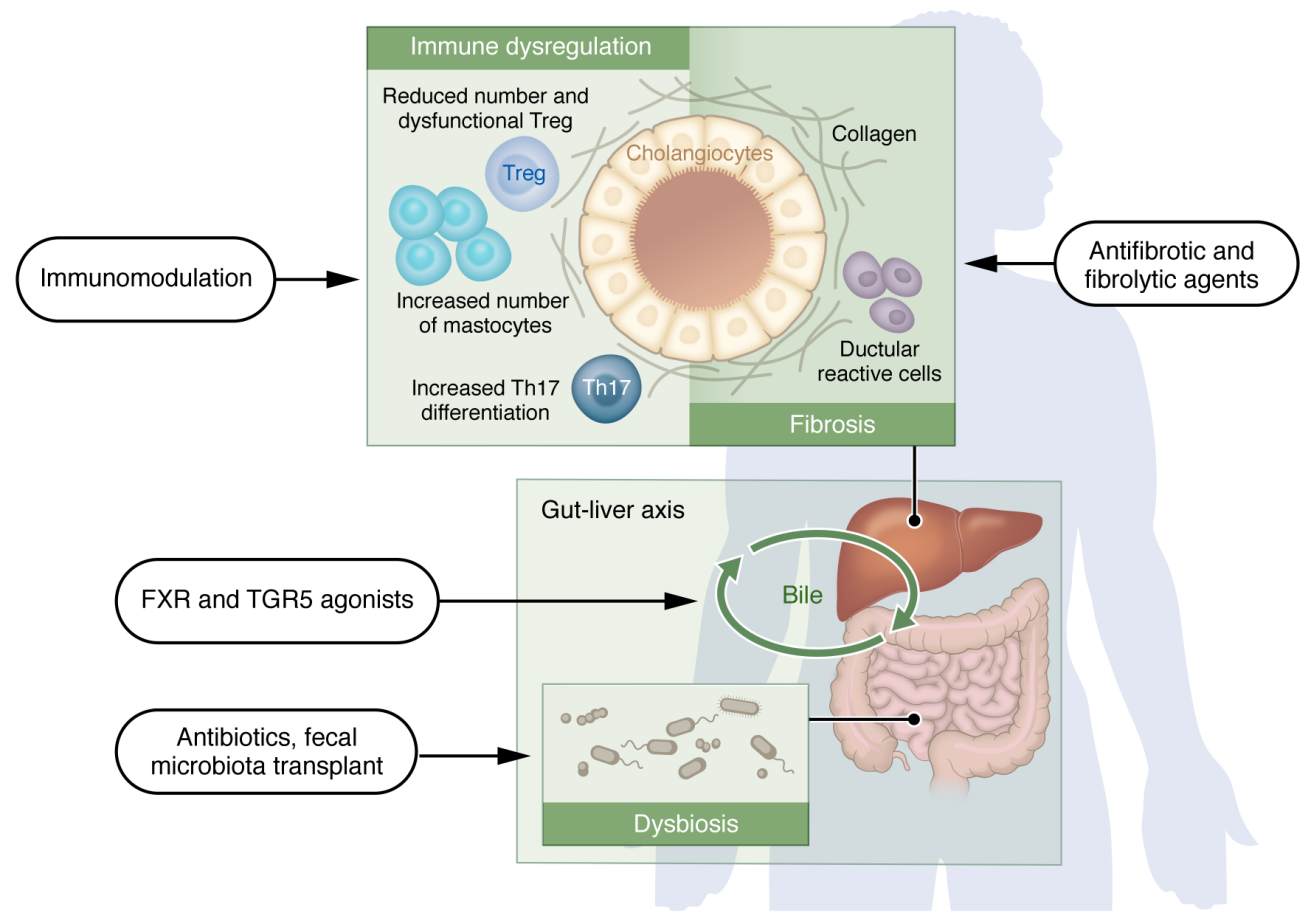
Schematic depiction of potential therapeutic areas for PSC. Emerging research suggests several possible strategies to treat PSC, including immunomodulation to reestablish “healthy” innate immune responses, manipulation of microbiota and the BA pool, as well as the development of novel antifibrogenic and fibrolytic approaches.

Primary sclerosing cholangitis (PSC) is an idiopathic cholestatic liver disease with progressive inflammation of the intra- or extrahepatic bile ducts, leading to segmental fibrosis and, ultimately, cirrhosis. It is considered a rare disease, with a prevalence of approximately 10–16 cases per 100,000 individuals (1). Since its earliest description in 1867 by CEE Hoffman, there have been hundreds of studies published regarding PSC’s natural history, diagnosis, and clinical features, including its strong association with inflammatory bowel disease (IBD). However, even with much-improved diagnostic technologies, PSC is still considered a clinically challenging disease. Patients with PSC generally have a higher risk for biliary complications and malignancies such as cholangiocarcinoma (2). Given the lack of effective pharmacotherapy, liver transplantation is still the main therapeutic option, however, there is a 25% of risk of disease recurrence in the graft. The precise etiology is still unknown but likely involves a combination of gut dysbiosis, altered bile acid composition, and unidentified “toxins” that may drive dysregulated innate immune responses in the liver. These factors ultimately culminate in stricturing, segmental biliary fibrosis. Synthesizing the knowledge gained from recent studies as outlined below could uncover the main driving pathways of this enigmatic disease. Our goal is not to provide a detailed review of recent findings but rather to focus on salient points that could be further explored for potential therapies (Figure 1).

## Genetic susceptibility studies

Several studies demonstrated an elevated risk of PSC among first-degree relatives (3). GWAS have described 23 loci associated with disease risk (4). Intriguingly, the genetic profile of PSC-associated IBD was distinct compared with Crohn’s disease or ulcerative colitis. Thus, PSC-IBD may represent a different entity, which is also supported by its clinical features (2). More recently, network-based drug-disease proximity analyses were performed to identify potential compounds that could be repurposed for PSC (5). The top-ranked drug in this study, denileukin diftitox, regulates immune tolerance by controlling Treg activity and could be a candidate agent for further studies. The same study suggested that ursodeoxycholic acid (UDCA), the mainstay of treatment for primary biliary cholangitis (PBC), may not be a genetically promising candidate drug for PSC. Accordingly, UDCA did not improve the course of PSC in recent clinical trials (6). Further understanding of the genetic architecture of PSC is of key importance. Improving risk stratification strategies, using multiomics analyses, and performing longitudinal studies of this population could be the next steps to identify potential candidate drugs.

## Molecular pathogenesis and potential therapeutic targets

In recent years, several studies have focused on defining gut microbial signatures and metabolites in PSC. These findings revealed reduced microbial diversity, as well as bacterial and fungal dysbiosis that was independent of the IBD-related microbiome signatures (7–9). Specifically, there was a marked increase in the *Veillonella* genus in patients with PSC-IBD when compared with both healthy controls and patients with IBD alone. To better evaluate a causative link between dysbiosis, intestinal permeability, and hepatobiliary effects, Nakamoto et al. transplanted fecal microbiota from patients with PSC into gnotobiotic mice and identified three bacterial strains (*Klebsiella pneumoniae*, *Proteus mirabilis*, and *Enterococcus gallinarum)* from their mesenteric lymph nodes (10). These were associated with hepatobiliary inflammation and a high Th17 response that was reversible with antibiotic treatment. These bacteria were also more prevalent in patients with PSC. More recently, a lytic phage cocktail was developed that targets *Klebsiella pneumoniae* and *Enterococcus gallinarum*. Administration of the cocktail improved liver inflammation and fibrosis in colonized specific pathogen–free mice (11). Awoniyi et al. studied protective versus detrimental bacterial species that can affect PSC outcomes and found that in the *Mdr2^–/–^* mice, short chain fatty acid–producing *Lachnospiraceae* species could have protective effects against *Enterococcus* *faecalis* and *E. coli* enterohepatic translocation, thereby exerting an antifibrotic effect in the liver. In patient cohorts, fecal *E*. *faecalis* and *Enterobacteriaceae* had a positive association with the Mayo risk score, while *Lachnospiraceae* showed a negative association with the score (12).

Despite these recent advances, clearly identifying a causative association between fecal microbiota and liver immune responses remains challenging. Further studies are needed to focus on gut epithelium–associated bacteria that could uniquely modify mucosal immune responses and affect disease severity.

Patients with PSC were also found to exhibit increased Th17 differentiation, which, as noted above, could be affected by the microbiota (10). Monocytes from patients with PSC have significantly increased IL-1β and IL-6 production (necessary for Th17 differentiation) compared with monocytes from healthy controls, and PSC patient PBMCs stimulated with *C*. *Albicans* produced significantly higher levels of IL-1β compared with healthy controls or patients with PBC (13). The first atlas of intrahepatic T cells in PSC demonstrated that naive CD4^+^ T cells have a propensity to develop into Th17 cells, exhibiting a predisposition to an effector function (14). Additionally, naive CD4^+^ T cells are less likely to differentiate into Foxp3^+^ Tregs. Studies also reported a reduced number of Tregs in PSC compared with PBC (15), and reduced Treg expansion was linked to upregulation of the IL-12 receptor (16). Stimulating Treg expansion with IL-2/anti–IL-2 immune complexes reduced the CD8^+^ T cell count and improved biliary injury and fibrosis in *Mdr2^–/–^* mice (17). GWAS revealed a correlation between the reduced expansion of Tregs and SNP of the IL-2 receptor α (15). Therefore, Treg dysfunction could have a key role in the pathogenesis of PSC. In a clinical trial using adoptive Treg transfer, Voskens et al. observed that ex vivo–expanded autologous Tregs from a patient with refractory ulcerative colitis and PSC resulted in a decrease in liver enzymes (47% decrease in alkaline phosphatase [ALP] by week 4) that lasted 4 weeks and returned to baseline by week 12 (18).

Mast cells (MCs) are tissue-resident immune cells that were observed to accumulate around the portal tracts in both human and animal models of PSC. Mice that were deficient in MCs exhibited decreased portal inflammation and ductular reaction, and these effects were linked to H2 histamine receptor signaling (19). MCs expressing farnesoid X receptor (FXR) played an important role in liver injury and regulation of bile acid (BA) levels through alteration of intestinal and biliary FXR/FGF15 signaling (20).

Another study showed that the FGF15/-19/FXR/CYP7A1 axis had a key role in modulating BA synthesis. In patients, suppressed BA synthesis portended a worse prognosis with low serum C4 levels (21). FXR agonism with cilofexor is currently being evaluated in a phase III trial.

TGR5 is a GPCR for primary and secondary BAs and was shown to have a protective role in biliary epithelial cells (BECs) by stimulating tight junction integrity. Reich et al. demonstrated TGR5 downregulation in PSC, and this contributed to a reactive BEC phenotype. Restoring TGR5 or increasing its level by norursodeoxycholic acid (norUDCA) resulted in improved liver enzymes and histology (22). Clinical trials focusing on norUDCA are currently ongoing.

Last, a significant amount of research has focused on the role of secretin and the secretin receptor (SR), which is only expressed on cholangiocytes within the liver. Recently, long-term administration of the SR antagonist (SCT 5-27) decreased ductular reaction and liver fibrosis in bile duct–ligated and *Mdr2^–/–^* mice through miR-125b and FoxA2 (23). Taken together, these preclinical studies provide a strong rationale to pursue clinical trials. Ongoing trials in this field are briefly summarized below.

## Clinical trials

Based on preclinical studies, FXR is an intriguing target for PSC studies. The results of the phase II and open-label extension trial of the FXR agonist cilofexor were promising, with improved liver enzymes and serum BA levels in noncirrhotic PSC patients (24). A large phase III trial (PRIMIS) is currently evaluating the safety and efficacy of the drug (ClinicalTrials.gov NCT03890120) (25). Phase III trials studying the effects of long-term simvastatin (NCT03041662), oral vancomycin (NCT03710122), and norUrsodeoxycholic acid (NCT03872921) are also currently underway. In addition, an open-label clinical trial evaluated the safety and efficacy of fecal microbiota transplantation in ten patients with concomitant IBD (NCT02424175). They found that ALP decreased by more than 50% in three patients, which correlated with improved microbiota diversity (26). Observational studies using vedolizumab (α4β7 integrin blocker) in patients with PSC-IBD showed no clear evidence of a biochemical response, although serum levels of ALP decreased by 20% or more in a subset of patients with a more aggressive phenotype of the disease (27).

## Summary and future possibilities

Despite tremendous efforts to define the key pathogenic features and therapeutic targets, effective medical therapy for PSC is still lacking. There are several obstacles that may impede faster progress. PSC animal models do not faithfully phenocopy the complex nature of human disease, thus translation may not be straightforward. We are now beginning to understand the early triggering events, such as the role of microbiota and mucosal immunity and their effects on innate immunity in PSC, and there is a significant effort to develop new biomarkers with acceptable sensitivity and specificity. High-throughput methods could be useful for biomarker discovery, cellular landscape analysis, and identification of novel pathways and networks related to the disease. The combination of single-cell and high-resolution spatial transcriptomics could advance the field by identifying new subsets of immune cells and their connection to other cell types or the extracellular matrix.

Furthermore, identification of the subsets of patients at high risk for disease progression or development of cholangiocarcinoma is critical. Improved surveillance involving a serum microRNA profile or AI-based technologies could help define predictive models (28).

With all the recent discoveries, effective medical therapy is expected to be available in the next few years. This could involve using combination therapies, considering genetic susceptibilities, and targeting different disease pathways such as those for immune dysregulation, fibrosis, and dysbiosis, and manipulating the microbiome.

## Version 1 09/01/2023

Electronic publication

## References

[1] Lazaridis KN, LaRusso NF (2016). Primary sclerosing cholangitis. N Engl J Med.

[2] Dyson JK (2018). Primary sclerosing cholangitis. Lancet.

[3] Bergquist A (2008). Increased risk of primary sclerosing cholangitis and ulcerative colitis in first-degree relatives of patients with primary sclerosing cholangitis. Clin Gastroenterol Hepatol.

[4] Ji SG (2017). Genome-wide association study of primary sclerosing cholangitis identifies new risk loci and quantifies the genetic relationship with inflammatory bowel disease. Nat Genet.

[5] Han Y (2023). Multitrait genome-wide analyses identify new susceptibility loci and candidate drugs to primary sclerosing cholangitis. Nat Commun.

[6] Rahimpour S (2016). A triple blinded, randomized, placebo-controlled clinical trial to evaluate the efficacy and safety of oral vancomycin in primary sclerosing cholangitis: a pilot study. J Gastrointestin Liver Dis.

[7] Sabino J (2016). Primary sclerosing cholangitis is characterised by intestinal dysbiosis independent from IBD. Gut.

[8] Kummen M (2017). The gut microbial profile in patients with primary sclerosing cholangitis is distinct from patients with ulcerative colitis without biliary disease and healthy controls. Gut.

[9] Lemoinne S (2020). Fungi participate in the dysbiosis of gut microbiota in patients with primary sclerosing cholangitis. Gut.

[10] Nakamoto N (2019). Gut pathobionts underlie intestinal barrier dysfunction and liver T helper 17 cell immune response in primary sclerosing cholangitis. Nat Microbiol.

[11] Ichikawa M (2023). Bacteriophage therapy against pathological Klebsiella pneumoniae ameliorates the course of primary sclerosing cholangitis. Nat Commun.

[12] Awoniyi M (2023). Protective and aggressive bacterial subsets and metabolites modify hepatobiliary inflammation and fibrosis in a murine model of PSC. Gut.

[13] Kunzmann LK (2020). monocytes as potential mediators of pathogen-induced T-Helper 17 differentiation in patients with primary sclerosing cholangitis (PSC). Hepatology.

[14] Poch T (2021). Single-cell atlas of hepatic T cells reveals expansion of liver-resident naive-like CD4^+^ T cells in primary sclerosing cholangitis. J Hepatol.

[15] Sebode M (2014). Reduced FOXP3(+) regulatory T cells in patients with primary sclerosing cholangitis are associated with IL2RA gene polymorphisms. J Hepatol.

[16] Schwinge D (2017). Dysfunction of hepatic regulatory T cells in experimental sclerosing cholangitis is related to IL-12 signaling. J Hepatol.

[17] Taylor AE (2018). Interleukin 2 promotes hepatic regulatory T cell responses and protects from biliary fibrosis in murine sclerosing cholangitis. Hepatology.

[18] Voskens C (2023). Autologous regulatory T-cell transfer in refractory ulcerative colitis with concomitant primary sclerosing cholangitis. Gut.

[19] Zhou T (2022). Mast cells selectively target large cholangiocytes during biliary injury via H2HR-mediated cAMP/pERK1/2 signaling. Hepatol Commun.

[20] Meadows V (2021). Mast cells regulate ductular reaction and intestinal inflammation in cholestasis through farnesoid X receptor signaling. Hepatology.

[21] Schneider KM (2021). Gut microbiota depletion exacerbates cholestatic liver injury via loss of FXR signalling. Nat Metab.

[22] Reich M (2021). Downregulation of TGR5 (GPBAR1) in biliary epithelial cells contributes to the pathogenesis of sclerosing cholangitis. J Hepatol.

[23] Wu N (2023). Prolonged administration of a secretin receptor antagonist inhibits biliary senescence and liver fibrosis in Mdr2^–/–^ mice. Hepatology.

[24] Trauner M (2023). Safety and sustained efficacy of the farnesoid X Receptor (FXR) agonist cilofexor over a 96-week open-label extension in patients with PSC. Clin Gastroenterol Hepatol.

[25] Trauner M (2023). PRIMIS: design of a pivotal, randomized, phase 3 study evaluating the safety and efficacy of the nonsteroidal farnesoid X receptor agonist cilofexor in noncirrhotic patients with primary sclerosing cholangitis. BMC Gastroenterol.

[26] Allegretti JR (2019). Fecal Microbiota transplantation in patients with primary sclerosing cholangitis: a pilot clinical trial. Am J Gastroenterol.

[27] Lynch KD (2020). Effects of vedolizumab in patients with primary sclerosing cholangitis and inflammatory bowel diseases. Clin Gastroenterol Hepatol.

[28] Hu C (2023). Predicting cholangiocarcinoma in primary sclerosing cholangitis: using artificial intelligence, clinical and laboratory data. BMC Gastroenterol.

